# Acute Administration of HIV-1 Tat Protein Drives Glutamatergic Alterations in a Rodent Model of HIV-Associated Neurocognitive Disorders

**DOI:** 10.1007/s12035-024-04113-8

**Published:** 2024-03-22

**Authors:** Brenna C. Duffy, Kirsten M. King, Binod Nepal, Michael R. Nonnemacher, Sandhya Kortagere

**Affiliations:** 1 Department of Microbiology and Immunology, Drexel University College of Medicine, Philadelphia, PA USA; 2 Center for Molecular Virology and Translational Neuroscience, Institute for Molecular Medicine and Infectious Disease, Drexel University College of Medicine, Philadelphia, PA USA; 3 Sidney Kimmel Cancer Center, Thomas Jefferson University, Philadelphia, PA USA

**Keywords:** Glutamate, HAND, HIV-1, NMDAR, Novel object recognition, mPFC, Transactivator of transcription, Working memory

## Abstract

HIV-1-associated neurocognitive disorders (HAND) are a major comorbidity of HIV-1 infection, marked by impairment of executive function varying in severity. HAND affects nearly half of people living with HIV (PLWH), with mild forms predominating since the use of anti-retroviral therapies (ART). The HIV-1 transactivator of transcription (Tat) protein is found in the cerebrospinal fluid of patients adherent to ART, and its administration or expression in animals causes cognitive symptoms. Studies of Tat interaction with the *N*-methyl-D-aspartate receptor (NMDAR) suggest that glutamate toxicity contributes to Tat-induced impairments. To identify changes in regional glutamatergic circuitry underlying cognitive impairment, we injected recombinant Tat86 or saline to medial prefrontal cortex (mPFC) of male Sprague–Dawley rats. Rats were assessed with behavioral tasks that involve intact functioning of mPFC including the novel object recognition (NOR), spatial object recognition (SOR), and temporal order (TO) tasks at 1 and 2 postoperative weeks. Following testing, mPFC tissue was collected and analyzed by RT-PCR. Results showed Tat86 in mPFC-induced impairment in SOR, and upregulation of *Grin1* and *Grin2a* transcripts. To further understand the mechanism of Tat toxicity, we assessed the effects of full-length Tat101 on gene expression in mPFC by RNA sequencing. The results of RNAseq suggest that glutamatergic effects of Tat86 are maintained with Tat101, as *Grin2a* was upregulated in Tat101-injected tissue, among other differentially expressed genes. Spatial learning and memory impairment and *Grin2a* upregulation suggest that exposure to Tat protein drives adaptation in mPFC, altering the function of circuitry supporting spatial learning and memory.

**Supplementary Information:**

The online version contains supplementary material available at 10.1007/s12035-024-04113-8.

## Introduction

Among the 38 million people living with HIV (PLWH), up to half experience HIV-associated neurocognitive disorders (HANDs). HAND is a collective term referring to cognitive impairment of varying severity in PLWH. HAND is inclusive of asymptomatic neurocognitive impairment (ANI), mild neurocognitive disorder (MND), and HIV-associated dementia (HAD). The cognitive domains impaired in HAND include learning, working memory, executive function, sensorimotor, and language. For a definitive diagnosis, the patient must exhibit impairment in two or more of these domains [1, 2]. The prevalence of HAD has decreased with the use of combination anti-retroviral therapies (CART); however, viral suppression by CART does not spare PLWH from cognitive impairment. ANI and MND each affect up to 30% of PLWH; thus, a broader population is affected by these moderate deficits [3]. Diagnosis with HIV can happen in early adulthood, and many new diagnoses occur in the population aged 13 to 34 [4]. Nonetheless, it is important to consider that a majority of PWH are aged 50 years or older, and age has been associated with cognitive decline in PWH [5, 6]. As there are no specific therapeutics for HAND and development of treatments is limited, it is critical to characterize the viral and host components driving mild forms of impairment to identify therapeutic targets.

Understanding the mechanisms driving cognitive impairment relies on models that recapitulate clinical findings, use relevant persistent viral components, and markers of CNS damage or alteration in HAND. The HIV-1 transactivator of transcription (Tat) is an early viral protein that persists in the cerebrospinal fluid of PLWH on CART [7]. This inherently disordered protein interacts with numerous host molecules, enabling some of its detrimental effects in the CNS. Animal models of HAND show that administration or expression of Tat protein recapitulates pathology and neurocognitive impairment [8, 9]. Tat-based models of HAND suggest that mechanisms underlying CNS damage involve modulation of neuronal and glial cell function to drive excessive excitatory signaling [10].

Tat protein affects neuronal signaling directly and indirectly, ultimately enhancing excitatory signaling and promoting neuronal death and synaptic loss [10, 11]. The effects of Tat on glial cells also contribute to neurotoxicity. Microglia are the primary cellular reservoir for HIV-1 in the CNS, and therefore are likely a significant source of circulating Tat protein [12–14]. Circulating Tat also stimulates microglia to release pro-inflammatory cytokines, potentially impacting neuronal loss and blood–brain barrier permeability [15, 16]. In addition to modulating inflammatory processes, Tat can promote microglial glutamate release, supporting maladaptation and damage to neurons as mediated by receptor signaling [17, 18]. Tat also modulates astrocytic functions, driving cytokine expression, ER stress, and limited glutamate uptake [19–23], providing another source of dysregulation. Astrocytes are the most abundant glial cells in the CNS and play a central role in supporting neuronal nutrient cycling, synaptic monitoring, and maintenance of the blood–brain barrier. Thus, the effects of Tat on astrocytes are equally as important as the effects on microglia in the development of HAND.

Various studies show that Tat protein promotes excitatory signaling in the brain, and alteration of glutamatergic signaling is hypothesized to play a central role in the pathology of HAND [24]. Tat transgenic mouse models suggest that ubiquitous or astrocyte-restricted expression of Tat enhances glutamate release in the cortex [25, 26], though region-specific effects are likely [26]. Both microglia and astrocytes play a significant role in regulating glutamate in the context of Tat neurotoxicity, dependent on glutamatergic receptors and transporters. Tat stimulates microglial release of pro-inflammatory cytokines, and glutamate by similar molecular signaling [27]. Tat protein suppresses the expression of the glial glutamate transporter EAAT2 [28]. Tat is known to interact directly with *N*-methyl-D-aspartate receptor (NMDAR) and potentiate receptor signaling [18, 29]. Thus, Tat alters multiple aspects of glutamatergic signaling, converging to increase extracellular glutamate levels.

In this study, we used recombinant Tat to evaluate its toxicity in mPFC and the associated cognitive impairment in adult rats. To understand the molecular mechanisms associated with Tat toxicity in mPFC, we performed RNA analyses of the tissue. Results from this study suggest that even at low doses representative of circulating Tat concentrations in patient CSF, Tat promotes changes in NMDA receptor (NMDAR) subunit transcription and impairments in spatial learning and memory.

## Results

### Tat-Injected Rats Exhibit Deficits in Spatial Object Recognition Tasks

The clinical symptoms of HAND encompass varying severity of impairment in cognitive functions, which rely on frontal cortex circuitry. To model the effect of HIV-1 Tat on executive function (learning and memory), recombinant Tat86 or saline was administered to mPFC of male rats; at one and two post-operative weeks, rats were tested on three behavioral tasks—Novel Object Recognition (NOR), Spatial Object Recognition (SOR), and Temporal Order (TO; also known as the Recency Task). This battery of tests relies on the tendency of rodents to recognize and explore novel objects or context.

A high dose of Tat86 (10 μg) was selected to assess toxicity in mPFC. In this study, one group of rats received intracerebral (IC) injection of saline (*n* = 6) and another group received injection of Tat86 (10 μg, IC; *n* = 6). Prior studies have suggested that Tat exerts excitotoxic effects via interaction with NMDA receptors when applied at doses in the micromolar range [29]. To assess this mode of toxicity, an additional group of rats was injected with NMDA (2 μg, IC; *n* = 4) as a positive control for NMDAR-mediated excitotoxicity.

**Fig. 1:**
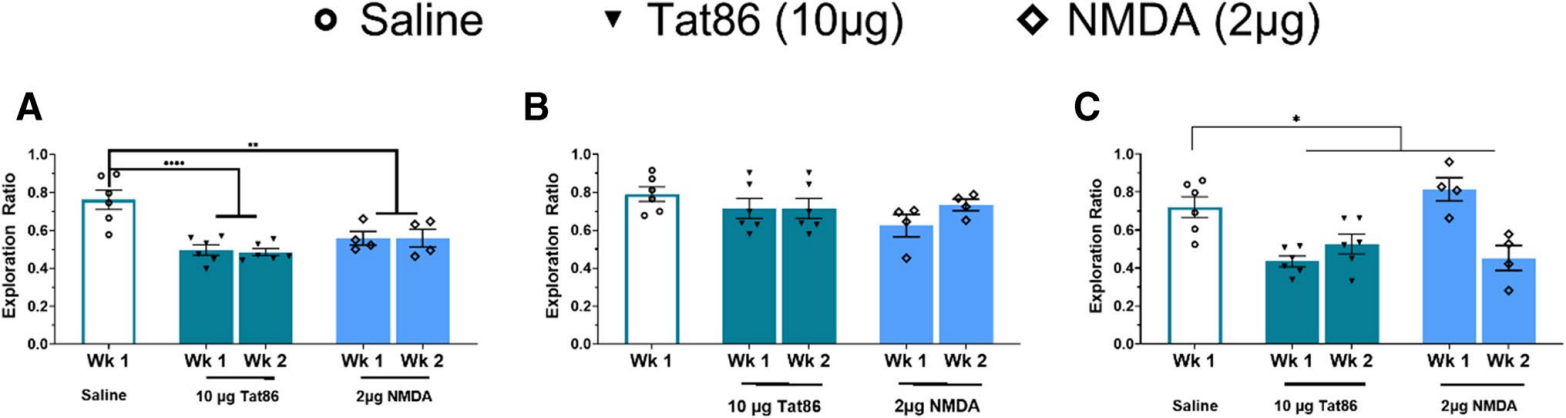
Injection of a high dose of Tat86 (10 μg) to mPFC resulted in impairment in spatial learning and memory, similar to NMDA-induced impairment. **A** In the SOR task, the Tat and NMDA groups exhibit lower mean ER when compared to the saline group. (Main effect of injection *P*adj < 0.0001 by mixed-effect two-way ANOVA with Dunnett’s correction). **B** In the NOR task, Tat- and NMDA-injected rats perform similarly to saline-injected rats, with similar mean ER values in all groups. **C** In the TO task, an overall treatment effect is detected (*P* < 0.05), and mean ER for the Tat group trends toward lower values relative to the saline group (*P*adj = 0.0671). Group means were compared by mixed-effect two-way ANOVA with Dunnett’s correction

The results of behavioral testing showed that saline-injected rats in the high-dose Tat86 cohort successfully completed all behavioral tasks (saline group mean exploration ratio (ER) ≥ 0.6, Fig. 1A–C). In contrast, the high-dose Tat group spent less time exploring the displaced object in SOR compared to the saline group (Tat group week 1 mean ER = 0.497, week 2 mean ER = 0.485; main effect *P*adj < 0.0001 by mixed-effect two-way ANOVA with Dunnett’s correction) (Fig. 1A). NMDA-injected rats exhibited similar reduction of mean ER when compared to saline-injected rats (NMDA week 1 mean ER = 0.558, week 2 mean ER = 0.559; main effect *P*adj < 0.01 by mixed-effect two-way ANOVA) (Fig. 1A).

The effects of Tat86 were not as apparent in TO and NOR tasks. No deficits were observed in either Tat- or NMDA-injected rats in the NOR task, as reflected by mean ER values greater than 0.6 in all groups (Fig. 1B). The Tat group exhibited mean ER > 0.6 at both 1- and 2-week timepoints, suggesting that circuitry involved in object recognition is not overtly altered by Tat injection. In the TO task, analysis by a mixed-effect 2-way ANOVA indicated a main effect of injection (*P* = 0.0438). Multiple comparison testing suggested that the mean ER for the Tat group trends toward lower values when compared to the saline group (*P*adj = 0.0671), with no support for a difference between the NMDA group and the saline group in the TO task (Fig. 1C). These results suggest that injection of high-dose Tat86 to mPFC correlated with impaired temporal memory; however, NMDA injection did not impair temporal memory (*P*adj = 0.8810). The combined results of TO and SOR suggest that high-dose Tat injection to mPFC is associated with overt impairment of circuitry underlying spatial memory and potential effects on temporal memory circuitry.

**Fig. 2:**
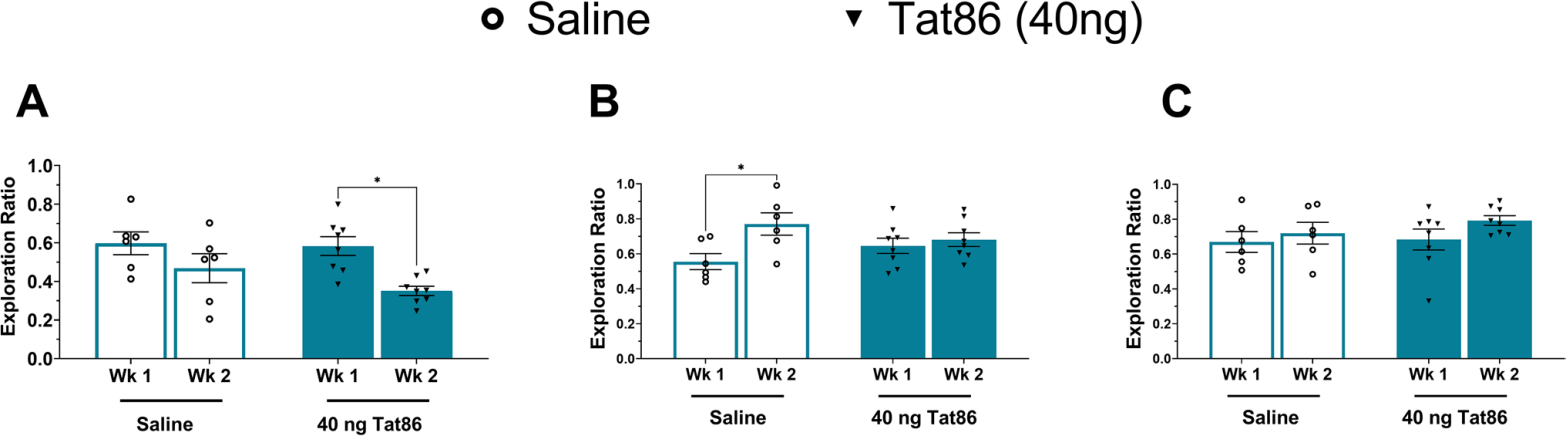
Injection of a low dose of Tat86 (40 ng) to mPFC is associated with impairment in the SOR task. **A** In the SOR task, analysis of mean ER values by 2-way ANOVA revealed a main effect of time (*P* < 0.05). Multiple comparisons revealed that rats in the cohort receiving injection of 40 ng of Tat86 spend less time exploring a displaced object at week 2 when compared to performance at week 1 (*Padj = 0.0314). **B** Analysis of the NOR task by 2-way ANOVA demonstrated a main effect of time (*P* < 0.05), and multiple comparison testing revealed that saline-injected rats exhibit higher mean ERs at week 2 compared to week 1 (*P < 0.05). **C** Analysis of the TO task revealed no significant effects or differences between groups. ERs were calculated based on the time spent exploring a displaced, new, or less familiar object (SOR, NOR, TO). Group means were compared by two-way ANOVA. *p < 0.05

In vivo, PLWH has been shown to have circulating Tat at levels as low as 200 pg/ml [7] and up to 40 ng/ml concentrations [30, 31]. As such, experiments were repeated with a low-dose Tat86 protein (40 ng), nearly 1000-fold lower than the high-dose group. Rats receiving this low-dose exhibited similar impairment as the high-dose group. While performance in the NOR and TO tasks was unaffected by injection of 40 ng of Tat86 when compared to the saline group, mean ER was reduced in Tat86-exposed rats tested on the SOR task (main effect of time, *P* = 0.0144 by two-way ANOVA; *P* = 0.0314 by Sidak’s multiple comparison test for Tat86 group) (Fig. 2A). This suggests that the clinically relevant dose of Tat86 exerts toxicity which specifically affects spatial learning and memory at the acute timepoint of 2 weeks (Fig. 2A). The results of NOR task also did not demonstrate changes in the Tat group; however, they indicate a significantly higher mean ER in saline-injected rats at post-operative week 2, when compared to saline-injected rats at week 1 (Fig. 2B). This suggests that there may be an acute effect of surgery at post-operative week 1 within the vehicle group, with improved object recognition following further recovery at the second post-operative week (Fig. 2B). The TO task results did not reveal distinctions between low-dose Tat and saline groups, suggesting that low-dose Tat did not impact temporal memory (Fig. 2C).

### NMDA Receptor Subunit Expression in mPFC is Altered Following Tat Administration

**Fig. 3:**
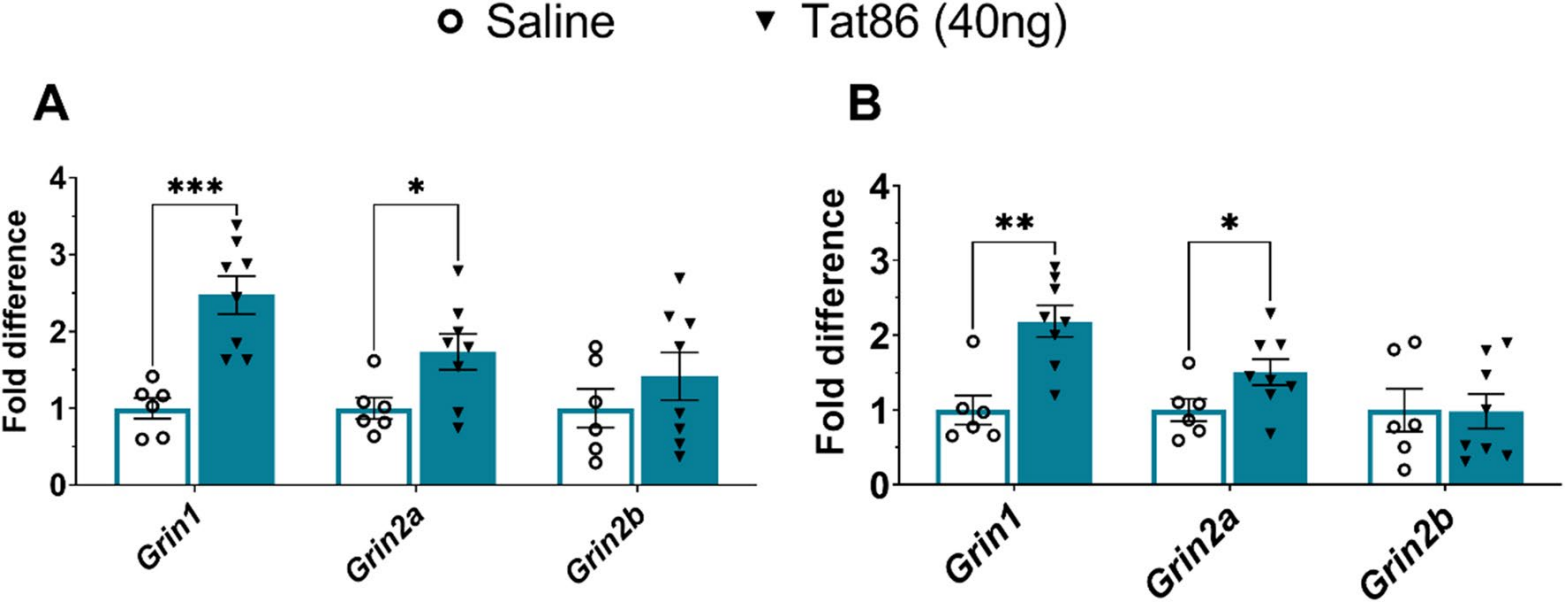
Transcripts of *Grin1* and *Grin2a* are increased in Tat86-lesioned mPFC and contralateral hemisphere. Gene expression was assessed by RT-PCR of mPFC RNA from rats that received unilateral injection of saline (vehicle; *n* = 6) or low-dose recombinant Tat86 (40 ng; *n* = 8). Transcripts for *Grin1* and *Grin2a* are increased in the lesioned hemisphere **(A)** and the unlesioned hemisphere **(B)**. Gene expression relative to the housekeeping gene β-actin was determined by the ΔΔ Ct method, stratified by injured hemisphere and controlled for a false discovery rate threshold of 5%. Fold change values for each gene were normalized against the average expression in saline-treated rats. Differences in fold change values were statistically assessed by multiple *t* test (*Padj < 0.05, **Padj < 0.01, ***Padj < 0.001)

Because the clinically relevant low dose of Tat86 was associated with impaired spatial learning and memory, mPFC tissue was collected from the low-dose group of the Tat86 cohort after the second week of behavioral testing. RNA was isolated from mPFC, and subsequently analyzed by RT-PCR to identify differences in transcription of genes of interest. To assess Tat-induced changes in glutamatergic pathways and pro-inflammatory signaling, transcripts were analyzed for genes encoding NMDA receptor subunits and pro-inflammatory cytokines. As the rats in this cohort received a unilateral injection of Tat86, normalized cycle threshold (Ct) values were stratified by injury to generate fold change values separately for the lesioned and unlesioned hemispheres. Fold change values for each gene were then normalized against the average expression in saline-treated rats. The resulting fold differences identify alterations in NMDA receptor subunit expression in mPFC in both hemispheres of Tat-injected rats (Fig. 3). Specifically, transcripts of *Grin1* which encodes the NR1 subunit of the NMDAR were increased in the lesioned mPFC of Tat-injected rats (*P*adj < 0.001), and in the contralateral mPFC (*P*adj < 0.01), relative to saline-injected rats (Fig. 3A, B). Transcripts of *Grin2a* which encodes the NR2A subunit of NMDAR were increased in the lesioned mPFC (*P*adj < 0.05) of Tat-injected rats and to a similar extent in the contralateral mPFC (*P*adj < 0.05) (Fig. 3A, B). In contrast, *Grin2b* which encodes the NR2B subunit exhibited no differences in transcript abundance when comparing Tat- and saline-injected groups. These results show that transcription of *Grin1* and *Grin2a*, encoding the NMDAR subunits NR1 and NR2A, is enhanced in mPFC of rats receiving a low dose of HIV-1 Tat86 protein.

**Fig. 4:**
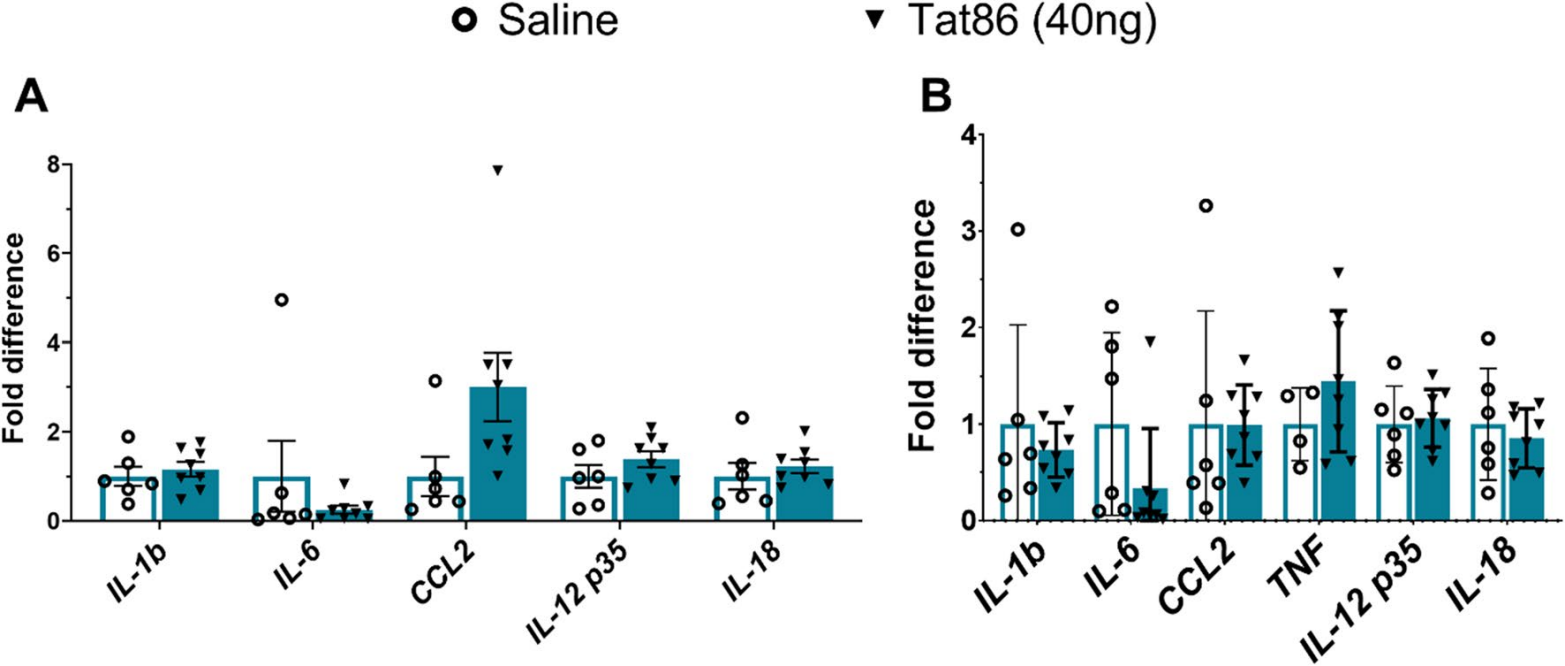
Cytokine and chemokine transcripts are not significantly altered in Tat-injected mPFC. RNA was extracted from mPFC of rats that received unilateral injection of saline (vehicle; *n* = 6) or low-dose recombinant Tat86 (40 ng; *n* = 8) for assessment of gene expression by RT-PCR. Gene expression relative to the housekeeping gene β-actin was determined by the ΔΔ Ct method, stratified by injured hemisphere and controlled for a false discovery rate threshold of 5%. Fold change values for each gene were normalized against the average expression in saline-treated rats. Differences in fold change values were statistically assessed by multiple *t* test. TNF was not consistently detectable in the lesioned mPFC samples, preventing quantification. Pro-inflammatory cytokines TNF and IL-1b and IL-6 were not significantly different in Tat-injected rats whether in the lesioned or contralateral mPFC. CCL2/MCP-1, IL12 p35, and IL18 also were not significantly different in the Tat group compared to the saline group. *y*-axis scales for panels **A** and **B** are different

Because exposure to Tat has previously been shown to influence the secretion of chemokines and pro-inflammatory cytokines, we also assessed transcript abundance for TNF, IL-1β, IL-6, CCL2 as well as IL-12 (p35) and IL-18 which are implicated in HIV infection. While transcripts were generally detectable, TNFα was below the limit of detection in several samples; therefore, the fold changes could not be calculated (Fig. 4). Overall, differences were not observed in any of the analyzed cytokines or chemokines, whether in lesioned or unlesioned tissue when comparing the Tat86 group against the saline group. Review of our RNA-seq data for interferon (IFN) signaling reveals 9 IFN-related genes (*Irf2bpl*, *Prkra*, *Ifngr1*, *Irf2bp1*, *Ifitm10*, *Ifi35*, *Ifi27l2b*, *Sting1*, *Irf3*) that are significant (*P*adj < 0.05) but do not pass the threshold for log2FC ≥ 1 or log2FC ≤  − 1, suggesting that interferons are not altered in the Tat-injected mPFC tissue. In contrast, comparison of our RNA-seq data against a manually curated Interferome database suggests that a number of genes expressed at distinct levels between Tat and saline-injected tissue (*P*adj < 0.05, log2FC ≥ 1 or ≤  − 1), are regulated by IFNs.

### Glutamate and GABA-related Genes are Upregulated in Tat101-injected mPFC

**Fig. 5:**
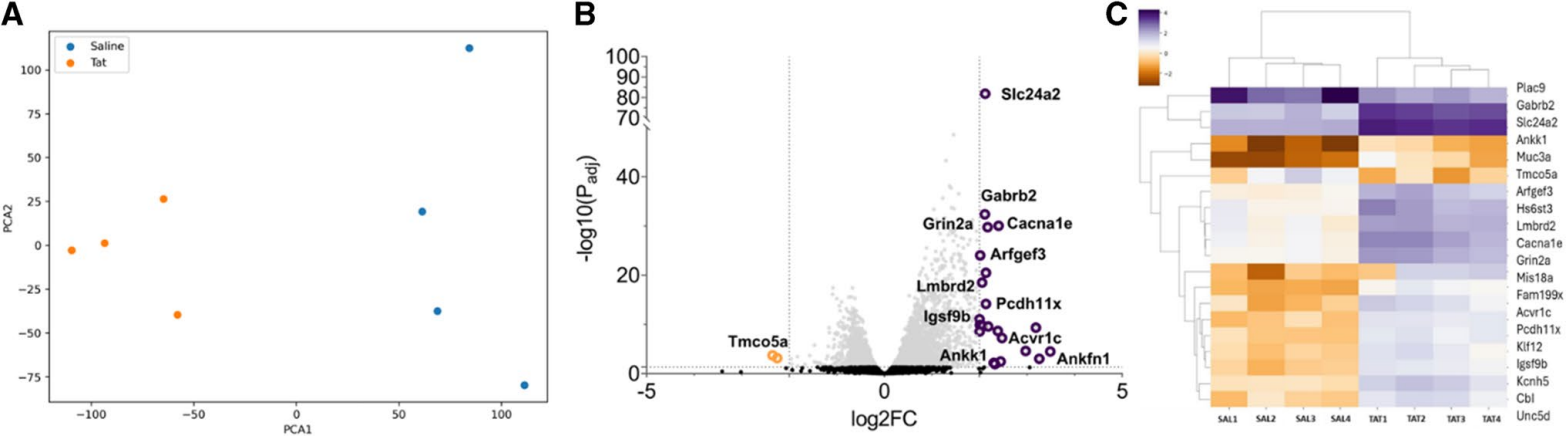
RNA-sequencing reveals differential expression of genes related to glutamatergic signaling, calcium channels, and intracellular signaling. RNA from the mPFC of rats receiving injection of Tat101 (40 ng, *n* = 4) or saline (*n* = 4) was sequenced by GeneWiz (Azenta Life Sciences, South Plainfield, NJ). Mapped reads were analyzed for differential expression using DESeq2 and filtered on *P*adj < 0.05 and Log2FC ≥ 2 or ≤  − 2. **A** Differential expression analysis indicates distinct Tat and saline groups (*n* = 4). **B** Several genes are upregulated in Tat-treated mPFC, suggesting increased glutamatergic, GABAergic, calcium, and other signaling pathways. **C** A clustered heatmap of the top 20 DE genes indicates clusters of genes that distinguish Tat and saline (SAL) groups by transcript expression

Because the clinically relevant low dose of Tat86 was associated with impaired spatial learning and memory and NMDA receptor expression, we sought to assess differential gene expression in an unbiased manner. To improve the relevance of our results to mechanisms underlying HAND in PLWH, the low dose of 40 ng was maintained; further, we chose to use the full-length Tat isoform, Tat101. Many studies on Tat neurotoxicity utilize a truncated 86 amino acid isoform of Tat protein (Tat86) to model HAND, as this truncated isoform maintains neurotoxic effects [10, 32]. However, the full-length Tat101 protein is more commonly found in clinical isolates [33]. Study of the Tat101 isoform may prove useful to accurately reflect mechanisms of HAND in PLWH, and to validate findings of prior studies with Tat86. Therefore, to assess differential gene expression, a cohort of male SD rats received intracerebral injection of Tat101 at a dose of 40 ng to mPFC in both hemispheres. Bilateral injection for this group was selected to allow for bilateral tissue collection, ensuring abundant RNA for sequencing. Brain tissue was collected 3 weeks after surgery, and mPFC was dissected for isolation of RNA for sequencing. Principal component analysis (PCA) was performed to detect outliers and examine differences between and within sample groups, revealing clear delineation of the Tat and vehicle groups (*n* = 4 per group) (Fig. 5A). Differential expression analysis with thresholds at *P*adj < 0.05 and − 2 ≤ log2FC ≥ 2 reveals 21 upregulated genes and 2 downregulated genes (Fig. 5B). The upregulated genes include *Grin2a*, validating the upregulation revealed in RT-PCR of Tat86-injected mPFC (Figs. 3 and 5B). In addition to this glutamatergic change, GABA receptor signaling components are upregulated in Tat-treated mPFC, including the GABA-A receptor subunit gene *Gabrb2*, and *Igsf9b.* Differential expression additionally implies the involvement of calcium signaling, with significant upregulation of *Slc24a2* and *Cacna1e* (Fig. 5B)*.* Other differentially expressed genes implicate cellular adhesion (*Igsf9b*, *Pcdh11x*), vesicular trafficking (*Arfgef3*), and protein–protein interactions (*Ankfn1* and *Ankk1)* (Fig. 5B, C). These differentially expressed genes in the Tat-treated rats in multiple gene clusters (Fig. 5C) suggest alterations in neurotransmission of both glutamate and GABA and their respective signaling pathways likely contributing to the observed neurocognitive impairment. Further analysis of our differential expression data by GO enrichment analysis (*P*adj < 0.05 and log2FC ≥ 1) indicates several pathways that are modified by Tat injection (Online Resource 4). The top 20 upregulated (Online Resource 4) pathways emphasize a handful of upregulated genes indicating upregulation of cytoskeleton and neurotransmitter regulation, among other pathways. We did not find any hits for the GO enrichment analysis (*P*adj < 0.05 and log2FC ≤  − 1) for the downregulated gene set.

## Materials and Methods

### Animals

Male Sprague–Dawley (SD) rats were acquired from Charles River Laboratories at 200–250 g body weight (7 to 8 weeks of age). All animal procedures were approved by the Institutional Animal Care and Use Committee of Drexel University. Rats were housed in the university laboratory animal housing facility on a 12-h light/dark cycle in a temperature-controlled room and given ad libitum access to standard chow and water.

### Recombinant Tat

Two isoforms of recombinant Tat were utilized in the study. Both isoforms were generated in *Esherichia coli* and purified to > 95% purity. The Tat86 isoform was obtained through the NIH AIDS Reagent Program, Division of AIDS, NIAID, NIH: from Dr. John Brady and DAIDS, NIAID. Tat86 was administered to rats in the behavior cohort, as this isoform has been utilized in most previously published studies to date. The Tat 101 amino acid isoform, administered in the RNA-seq cohort, was obtained from ImmunoDx (item no. 1032: Recombinant Tat HIV-1 MN). Both isoforms contain the domain responsible for NMDAR-mediated toxicity [10, 32].

### Stereotaxic surgery

Rats were acclimated for 1–2 weeks before undergoing stereotactic surgery and were at least 10 weeks old when they were chosen for surgery. Sprague–Dawley rats (*n* = 38) underwent stereotactic surgery to administer sterile 0.9% saline (vehicle) or HIV-1 Tat protein (Tat86aa, 40 ng or 10 μg, NIH AIDS Reagent Program; or Tat101aa, 40 ng, ImmunoDx) by intracerebral injection as previously described [34]. Briefly, rats were anesthetized with isoflurane, an incision was made at midline, and bregma was located. Craniotomies were drilled at mPFC (stereotaxic coordinates A/P + 3.0, M/L ± 0.7, D/V − 3.0; Paxinos and Watson [35]). Either sterile saline (sham control) or HIV-1 Tat protein formulated in sterile saline (2 μl) was injected at a rate of 0.5 μl per minute using a Hamilton syringe. The syringe was left in place for 2 min before slowly retracting the syringe, cleaning the skull, and closing the incision site with sterile sutures. For behavioral studies, three cohorts of rats were used and received saline (*n* = 12), Tat86 (40 ng, *n* = 8; 10 μg, *n* = 6), or NMDA (2 µg, *n* = 4). A separate cohort of rats was used for RNAseq experiments and received either saline (*n* = 4) or Tat101 (40 ng, *n* = 4).

Post behavioral studies, rats were euthanized and mPFC tissue was extracted for RT-PCR experiments. Rats from the RNA-seq cohort were euthanized 20 days post-surgery (time point chosen to match the behavioral cohort); mPFC region was extracted and stored in RNA-Later for RNA extraction.

### Behavioral testing

Male SD rats were allowed to recover from surgery for 1 week before performing behavioral tests. Behavioral testing consisted of three tasks: novel object recognition (NOR), spatial object recognition (SOR), and temporal order (TO) tasks. All rats completed the three tasks sequentially over 5 days, at both one and two post-operative weeks. All behavioral tasks were performed as previously described [36]. Briefly, rats were placed in a plastic chamber (60 cm L × 40 cm W × 30 cm H) with interior spatial cues for 2 days of acclimation prior to testing. Rats were returned to the chamber for each task, to explore sets of objects.

In the NOR task, rats were first exposed to two identical objects before a test trial, where they were presented with one object from the prior trial, and a new object. The SOR task, testing spatial learning and memory, is similar to NOR except one object from the trial period is displaced in the testing period. Finally, in the TO task, rats were allowed to explore a pair of identical objects in trial 1 as in NOR and SOR. In subsequent trials, rats were allowed to explore a pair of novel objects (trial 2), and finally encountered one object each from trial 1 and trial 2. For all tests, the amount of time spent exploring each object in the test trial was recorded, and the total exploration time was calculated. These times were then used to calculate the exploration ratio (ER) (Online Resource 1). An ER of 0.6 was the criterion for successfully completing each task, as expected in non-lesioned rats that spend more time to explore the novel, displaced, or less recent object. Mean ERs were compared by two-way ANOVA or mixed-effect two-way ANOVA with Dunnett correction.

### RNA extraction

RNA was isolated from mPFC tissue 3 to 5 weeks post-surgery, from the following groups: saline (*n* = 14), Tat86 (40 ng, *n* = 8), and Tat101 (40 ng, *n* = 8) using the TRIzol-chloroform method. Briefly, mPFC tissue was homogenized in TRIzol (Invitrogen) before adding chloroform and inverting to separate the organic and aqueous phases. Nucleic acids were precipitated from the aqueous phase via a series of washes with isopropanol and ethanol. The nucleic acid pellet was then treated with TURBO DNase (Invitrogen) to isolate RNA. RNA was purified by addition of sodium acetate, glycogen, and 100% ethanol, followed by multiple washes in 75% ethanol. Finally, the RNA pellet was dried and resuspended in nuclease-free water. RNA quantity and quality were assessed by NanoDrop, and total RNA was stored at − 80 °C.

### Reverse transcriptase-polymerase chain reaction (RT-PCR) (Tat86 cohort)

RT-PCR was carried out as previously described [37]. Briefly, RNA (250 μg) from mPFC was used to synthesize cDNA with the High-Capacity cDNA Reverse Transcription Kit (ThermoFisher). cDNA was then amplified by PCR using the QuantStudio 6 Flex thermal cycler (ThermoFisher) to assess relative quantities of cDNA for the following genes: *Grin1*, *Grin2a*, *Grin2b*, *IL-1β*, *TNF*, *IL-6*, IL-12 p35, and *IL-18* (Online Resource 2 for primer sequences). Gene expression relative to the housekeeping gene β-actin was determined by the ΔΔ Ct method, stratified by injury, and controlled for a false discovery rate threshold of 5%. Following normalization of fold change values for each gene against the average expression in saline-treated rats, relative expression was analyzed by multiple *t* test with Holm-Sidak correction.

### RNA sequencing (Tat101 cohort)

RNA (2 μg) extracted from mPFC of rats receiving injection of Tat101 or saline was aliquoted from each sample for sequencing by GeneWiz (Azenta Life Sciences, South Plainfield, NJ). RNA-seq reads were provided as FASTQ files, mapped using Salmon [38], and analyzed for differential expression using DESeq2 in R Studio [39]. DESeq2 output is available in Online Resource 3.

### Gene Ontology (GO) Enrichment Analysis

GO enrichment analysis (Online Resource 4) was done with a Cluster Profiler package [40] in the R-program version 4.3.1. The Cluster Profiler package and the organism-gene database for rattus norvegicus [41] (org.Rn.eg.db) were installed from the Bioconductor site [42]. The list of the upregulated genes was selected with a *P*adj value ≤ 0.05 and log2fold change value ≥ 1. The list of downregulated genes was selected with the *P*adj value ≤ 0.05 and log2fold change values ≤ 1 to provide added context to the discussion of pathway enrichment. The 20 most significant biological processes were plotted (Online Resource 4).

Figures were prepared in GraphPad Prism (Figs. 1, 2, 3, 4 and 5B) and Python (Fig. 5A, C Online Resource 4).

## Discussion

The current studies were designed to investigate the glutamatergic mechanisms underlying HIV-1 Tat-mediated toxicity in a rat model of HAND. Prior studies have clearly demonstrated that Tat drives neurotoxicity in cell culture and cognitive impairment in animal models of HAND. Importantly, the physical interaction of Tat with NMDARs is critical for Tat’s induction of neuronal death. To examine Tat-induced glutamate toxicity in vivo and its impact on cognitive function, we administered a high or low dose of Tat to mPFC and examined performance on learning and memory tasks, and changes in gene expression. The results demonstrate that acute administration of HIV-1 Tat86 to mPFC at both a high and low dose corresponds with deficits in spatial learning and memory. Additionally, low-dose Tat86 and Tat101 caused changes in NMDAR subunit expression in the mPFC.

The rats injected with high dose of Tat86 (10 μg) specifically exhibit a lower mean ER in the SOR task when compared to saline-injected rats, as does the NMDA group, indicating a lower proportion of time exploring the moved object (Fig. 1A). Analysis of performance in NOR and TO shows no statistically significant differences observed between the Tat and saline groups. Though the high dose (10 μg) shows a trend toward impairment on TO, the more relevant low dose (40 ng) indicates no impairment on the TO task. Additionally, the NMDA-injected group did not exhibit significantly different mean ERs when compared to the saline-administered rats suggesting that overt glutamatergic toxicity did not affect NOR or TO behavior. The lack of NMDA-mediated toxicity may be impacted by the unmatched size of the NMDA group compared to the saline and Tat groups. Further, the relatively low group sizes may limit detection of differences in performance on the other behavioral tests. In particular, the high-dose Tat86 cohort suggests some effect on TO task that may be limited by small group size. Nonetheless, NOR and TO tasks are mediated by circuitry distinct from that underlying spatial learning and memory, and thus, the results suggest a circuit- or subregion-specific susceptibility to Tat-mediated glutamate toxicity. Performance in the SOR task relies on mPFC and the hippocampus, with some contribution from perirhinal cortex [43]. Temporal order/recency memory is also dependent on hippocampus [43–45]; additionally, TO and NOR performance are more dependent on perirhinal cortex [46, 47], whereas mPFC-hippocampus circuitry is most critical for spatial object recognition [48, 49]. The reduced performance of Tat86-injected rats in SOR taken together with no significant differences observed in NOR or TO implies that specific correlates of spatial learning and memory are affected by Tat, sparing mPFC-perirhinal circuitry involved in NOR and TO. Additionally, the impaired SOR performance observed in the NMDA-injected group suggests that Tat86 impacts circuitry underlying spatial learning and memory via NMDAR signaling. Thus, based on the findings of the current study, mPFC projections to CA1 and/or CA3 of the hippocampus may be particularly altered by Tat protein in mPFC.

Prior studies consistently demonstrate that exposure to Tat results in spatial learning deficits via glutamatergic mechanisms. Tat is known to impair spatial learning and memory including radial arm maze [50], likely via NMDAR signaling [51]. Tat expression in transgenic animals also impairs performance in the Morris Water Maze [52]. Spatial learning and memory deficits are also observed long-term following neonatal Tat exposure [53, 54]. These findings are in accordance with the abundant evidence of Tat potentiating NMDAR signaling. NMDAR potentiation in mPFC-hippocampal circuitry is suggested to contribute to impairment in mPFC-dependent tasks [55], and NMDAR signaling in the hippocampus is required for spatial learning and memory [56]. These studies additionally suggest that Tat reduces long-term potentiation [50, 51], offering another mechanism to examine in future studies. However, Tat impairment of spatial learning and memory is largely shown by injection to the hippocampus. It therefore remains critical to further investigate whether mPFC exposure to Tat leads to compensatory mechanisms or adaptation in specific subregions to affect connectivity to hippocampus.

To follow up on the consistent impairment of spatial learning and memory by both high and low doses of Tat86, and similar effects between high-dose Tat86 and NMDA treatment, we hypothesized that glutamatergic signaling in the mPFC may be altered in Tat-injected rats. It is also well established that cellular exposure to Tat stimulates pro-inflammatory signaling—particularly from activated microglia. Thus, we assessed transcript abundance for cytokines and chemokines as well as *Grin1*, *Grin2a*, and *Grin2b* which encode NMDA receptor subunits NR1, NR2A, and NR2B. While the NMDA receptor may also utilize NR2C or NR2D subunits, these are unlikely to be expressed in mPFC as they are restricted to early developmental time periods and/or specific cell populations [57]; NR2A is ubiquitously expressed, and NR2B is abundant in the frontal cortex [58].

Considering the importance of NMDARs in LTP and subsequent relation to spatial learning and memory, our findings regarding the upregulation of *Grin1* and *Grin2a* transcripts in mPFC (Figs. 3 and 5a) are of particular interest. Moreover, *Grin1* and *Grin2a* transcripts are also enriched in the contralateral hemisphere of Tat86-injected rats, which was not directly lesioned in the Tat86 cohort. These results suggest an effect beyond the site of injection, which may be due to spread of the injection or cross-talk within mPFC.

While Tat is known to potentiate NMDAR signaling, alteration of NMDAR expression is not well established. One observational clinical study suggests that overall NMDAR expression is reduced in the frontal cortex of individuals with AIDS and HAD, whereas we observe increased *Grin1* and *Grin2a* transcript expression specifically in rat mPFC. In vitro and in vivo models provide varying results regarding the effects of Tat on NMDAR expression. In an ethanol withdrawal model, Tat administration to CA1 of hippocampus does not alter NMDAR density, as assessed by MK801 binding in tissue lysates [51]. Primary rat hippocampal neurons treated with an LTP blocker following Tat-induced synaptic loss exhibited NMDAR reactivity in new postsynaptic densities, suggesting that synaptic loss caused by Tat may specifically affect NMDARs [59].

Other studies indicate that Tat exerts subunit-specific effects on NMDARs in vitro and in vivo. Selective antagonism of GluN2A-containing NMDARs can prevent Tat-induced synapse loss, whereas GluN2B antagonism limits cell death [60]. Further, GluN2B-specific antagonism *after* Tat-induced synaptic loss stimulates synapse replacement in vitro while rescuing Tat-induced dendritic spine loss and restoring fear conditioning in vivo [60, 61]. This study suggests that GluN2A stimulation enables neuronal pro-survival signaling, whereas antagonism of GluN2B containing NMDARs in the presence of Tat may limit pro-death signaling to enable the recovery of NMDAR expressing postsynaptic densities. The subunit-specific findings likely contribute to the observed behavioral outcomes; differential recruitment of GluN2A and GluN2B affects long-term potentiation in the hippocampus; thus, the observed upregulation of *Grin1* and *Grin2a* may particularly alter the effects of mPFC afferents on hippocampal LTP to promote impairment in SOR [62]. In addition, knockout studies suggest that the NR1 subunit in CA1 pyramidal cells of the hippocampus is required for intact object recognition [63], while other studies suggest that object recognition tasks increase NR1 and NR2A expression [64]. These findings suggest that increased expression of NR1 and NR2A in the hippocampus supports memory consolidation, and various studies suggest that expression of NR1 and NR2A subunits is increased following induction of long-term potentiation [65]. These studies may support our observation of increased expression of NR1 transcripts in the Tat group in combination with intact NOR performance; however, prior studies are specific to hippocampal expression. Specific findings regarding NMDAR subunit involvement in learning and memory in mPFC are contradictory. Whereas hippocampus exhibits increased NR1 and NR2A expression reflective of LTP following novel environment habituation, subunit expression in mPFC is not altered [66]. However, in the context of spatial tasks, NR1 and NR2A are increased in mPFC synapses following radial maze training, followed by further NR1 increase and NR2B increases after performance in additional tests [67].

Glutamatergic signaling in the mPFC is suggested to support amyloid beta-induced impairment in object recognition, as post-training NMDAR antagonist administration rescues performance in the object recognition test [68]. In contrast, systemic administration of NMDAR antagonist MK801 induced an animal model of schizophrenia involving spatial memory deficits [69]. Similarly, chronic MK801 induction of schizophrenia is suggested to limit LTP in the mPFC-hippocampus circuitry [70].

It is important to highlight that a number of studies suggest that behavioral testing alters gene expression. Memory consolidation following behavioral tasks may alter NMDAR subunit expression specifically [64, 65]. It is therefore possible that behavioral testing in our Tat86 cohort is contributing to the observed increases in *Grin1* and *Grin2a* expression. Notably, our RNA-seq study was carried out in animals that did not perform behavioral testing providing additional context. While *Grin2a* upregulation is confirmed in the RNA-seq data, suggesting that this is truly an effect of Tat exposure in mPFC, *Grin1* upregulation was not upheld. Thus, future studies should consider the potential effects of behavior training on *Grin1* and other NMDA subunit expression to understand if exposure to Tat protein alone influences gene upregulation.

Analysis of Tat101-exposed mPFC tissue by RNA-seq confirmed the upregulation of *Grin2a* and reveals other potential mechanisms of Tat toxicity. One of the most prominent upregulated genes in Tat-injected rats is *Slc24a2*, which encodes the calcium-sodium exchanger NCKX2 [71]. NCKX2 is widely expressed in the brain and suggested to play a critical role in axonal Ca^2+^ ion regulation, synaptic plasticity, and learning and memory [72, 73]. Given the importance of calcium influx to Tat toxicity through NMDAR potentiation [10, 17, 74], the upregulation of *Slc24a2* in Tat-injected mPFC may occur in response to Tat-mediated neuronal calcium influx. Other upregulated genes highlight the central role of calcium in Tat-induced toxicity in our study—*Cacna1e*, encoding a high voltage activated subunit Cav2.3 which plays a role in synaptic plasticity [75, 76]. Interestingly, two GABA signaling-related genes are also upregulated. While *Igsf9b* and *Gabrb2* have not specifically been reported in prior studies of HAND, GABAergic changes have been noted in Tat models of HAND [25, 26]. Tat-transgenic mice have been reported to exhibit PFC-specific reduction of Syt2 and gephyrin, but increased GAD67, indicating specific molecular changes rather than overall reduction of GABAergic signaling [77]. The upregulation of *Igsf9* and *Gabrb2* revealed by our RNA-sequencing analysis emphasize this variable effect of Tat on different GABAergic molecules. The additional finding of *Ankk1* and *Ankfn1* upregulation supports an overall effect of synaptic remodeling. Upregulation of these ankyrin-repeat containing molecules along with receptor subunit changes (*Grin2a, Gabrb2*) and other synapse-related adhesion molecules (*Igsf9b, Pcdh11x*) suggests the potential for synaptic remodeling after Tat injection to mPFC. Additional assessment of the differentially expressed genes at the threshold of − 2 ≤ log2FC ≥ 2 against the Interferome database [78] suggests interferon regulation of *Slc24a2*, *Mis18a*, and *Ankfn1*. By lowering the threshold for − 1 ≤ log2FC ≥ 1, many additional genes from Tat-treated mPFC match the database of interferon-regulated genes, including *Adamts9*, *Aif1*, *Cxcl10*, *Egfr*, *Fgf2*, *Gabrg3*,* Map3k1*, *Ppargc1b*, *Slc26a2*, and *Cldn1*. Interestingly, comparison of our DE genes against a published rat blood–brain barrier transcriptome [79] revealed no hits, and searching our gene list for genes associated with gap junctions, tight junctions, and established modes of Tat-mediated barrier disruption [80] similarly revealed few matches. However, a number of the aforementioned genes may play roles in cellular adhesion or blood–brain barrier permeability (namely *CXCL10*, *Cldn1*). While these genes are not featured in the differentially expressed genes in our RNA-seq results (Fig. 5) due to strict threshold limits, they can be useful candidates for future investigation. Our current results may also be limited by the bulk RNAseq approach, whereas single-cell RNA-seq may provide a refined understanding of cell-type specific Tat toxicity in future studies.

Additional analysis of our differentially expressed genes by GO analysis for pathway enrichment suggests broader changes beyond glutamate and GABAergic signaling (Online Resource 4). The top 20 significantly upregulated pathways identified by GO analysis highlight axon development and transport, cytoskeleton and filament organization, and glutamatergic transmission. Notably, axonal and synaptic transport has previously been implicated in Tat toxicity [81, 82]. MAP2 is commonly indicated across multiple pathways, and the loss of MAP2-positive dendrites has been reported in HIV-1-exposed neurons [83, 84]. In pathways related to cytoskeleton organization, axon and projection guidance, Mef2c and Cntn1 (contactin-1) stand out for their existing relation to synaptic plasticity and memory [85, 86], with Mef2c particularly having suggested protective roles [87–89]. The upregulation of neurotransmitter and glutamatergic regulation highlight *Syt1* and *Slc1a2* which have previous associations with Tat-induced toxicity [23, 90], providing some validation to our model. Particularly, *Slc1a2* (encoding the glutamate transporter GLT-1) within neurotransmitter regulation pathways recapitulates the relevance of glutamate dysregulation and excitotoxicity in Tat-exposed mPFC.

GO analysis did not indicate significantly downregulated pathways at the threshold of *P*adj < 0.05 and log2FC ≤  − 1. Below this threshold, pathways appear emphasizing ribosomal and translation processes, and importantly mitochondrial pathways including metabolic and oxidative stress responses. These pathways are notable for their involvement in neurodegenerative diseases and cognitive functions and may be important candidates for future studies with higher group sizes and statistical power, as mitochondrial dysfunction and mitophagy are important factors in HAND [91] and in Tat toxicity in neurons and glial cells [92–94].

Overall, the results of GO enrichment analysis support our findings in RT-PCR while also broadening the scope our findings to recapitulate some of the known hallmarks of neuronal and glial toxicity mounted in HAND and Tat-based models of the disease state.

Some transcriptomic analyses of HAND models recapitulate some of these expression changes. Animal models suggest that chronic exposure to Tat is correlated with upregulation of immune and inflammatory genes in the hippocampus [95, 96], while our RNA analyses did not highlight this, the result may be limited by the acute injection of Tat weeks before tissue collection. Notably, human studies utilizing RNA sequencing demonstrate that glutamate receptor subunits and transporters, ion channels, and immune signaling aspects are upregulated in brain tissue from PLWH [97]. The similarity between this clinical study and our differentially expressed genes suggests that the use of Tat101 at a clinically relevant isoform provides an accurate model of HAND-related transcriptional changes. However, distinct region-specific transcriptional changes, mechanisms of Tat-induced gene modulation, and the relationship between multiple neurotransmitter and ion signaling changes must be further investigated.

## Conclusion

This study demonstrates that HIV-1 Tat protein administered to mPFC specifically impairs spatial learning and memory in the SOR task and thus spatial learning and memory. In the same set of rats, we have demonstrated a change in NMDAR subunit transcription in Tat-injected mPFC. The results of the Tat101 cohort in RNA-sequencing analysis support the glutamatergic alteration seen in the Tat86 behavioral cohort and reflect previously demonstrated mechanisms of Tat toxicity in the brain—namely, calcium signaling and ion transport. Thus, we hypothesize that heightened glutamatergic transmission contributes to cognitive impairment in Tat-induced toxicity, reflective of clinical symptoms of HAND.

The findings of this study are limited by the application of an acute injection of Tat. While other studies have shown that a single dose at early timepoints can affect cognitive function in the long term, the use of recombinant protein introduces the possibility of early degradation of Tat. Various intracellular host factors exert opposing effects on the rate of Tat degradation [98–100], and extracellular degradation of Tat is not well characterized, limiting our ability to distinguish when the behavioral and molecular changes were established and how Tat protein degradation may have affected the experimental outcomes. In particular, the lack of differences in cytokine expression (Fig. 4) may be affected by degradation of Tat and transient cytokine release by glial cells. The timepoint of tissue collection approximately 3 weeks after a low-dose Tat injection and relatively low group numbers may have also limited the detection of these cytokines and chemokines. Regardless, the acute administration of a single dose of recombinant Tat did induce cognitive impairment and NMDAR subunit gene expression changes. Further studies will need to confirm whether these gene expression changes detected in our study impact protein expression. It will also be critical to recapitulate these in studies with older animals and/or chronic exposure to Tat. Most PWH in the USA are 50 years of age or greater [101], and age has been associated with cognitive decline in PWH [5, 6]. Therefore, studies in older animals and/or chronic exposure over a longer time period will be important to validate our findings at these disease-relevant ages. It will then be important to model chronic low-dose exposure to Tat secreted from viral reservoirs to more accurately model dose and temporal effects on these outcomes.

While prior studies support NMDAR subunit alterations and glutamatergic mechanisms of Tat toxicity, there are few novel therapeutic targets for treatment of HAND. Therapeutics may prove beneficial in studies of Tat toxicity in the CNS, including those targeting NMDAR directly and those affecting NMDAR activation by reducing extracellular glutamate. By limiting the activation of excess NMDARs via high extracellular glutamate, novel therapeutic options could promote neuronal and synaptic survival as well as improved cognitive function.

## Supplementary Information

Below is the link to the electronic supplementary material.

Supplementary file1 (XLSX 12 KB)

Supplementary file2 (XLSX 10 KB)

Supplementary file3 (CSV 4951 KB)

Supplementary file4 (PNG 953 KB)

## Data Availability

All data supporting the findings of this study are available within the paper and its Supplementary Information includi ng Online Resource 1 (behavior test criteria), Online Resource 2 (RT PCR primers), Online Resource 3 (raw data from RNA seq), and Online Resource 4 (GO enrichment analysis results). The data for behavioral studies can be easily visualized from Figures 1 and 2 for individual rats, and raw datasets will be shared if requested.
