# Supplementary figures and images for: Acute Administration of HIV-1 Tat Protein Drives Glutamatergic Alterations in a Rodent Model of HIV-Associated Neurocognitive Disorders
Source: Mol Neurobiol. 2024 Mar 22;61(10):8467–80. doi: 10.1007/s12035-024-04113-8 (PMC11415472; doi:10.1007/s12035-024-04113-8)

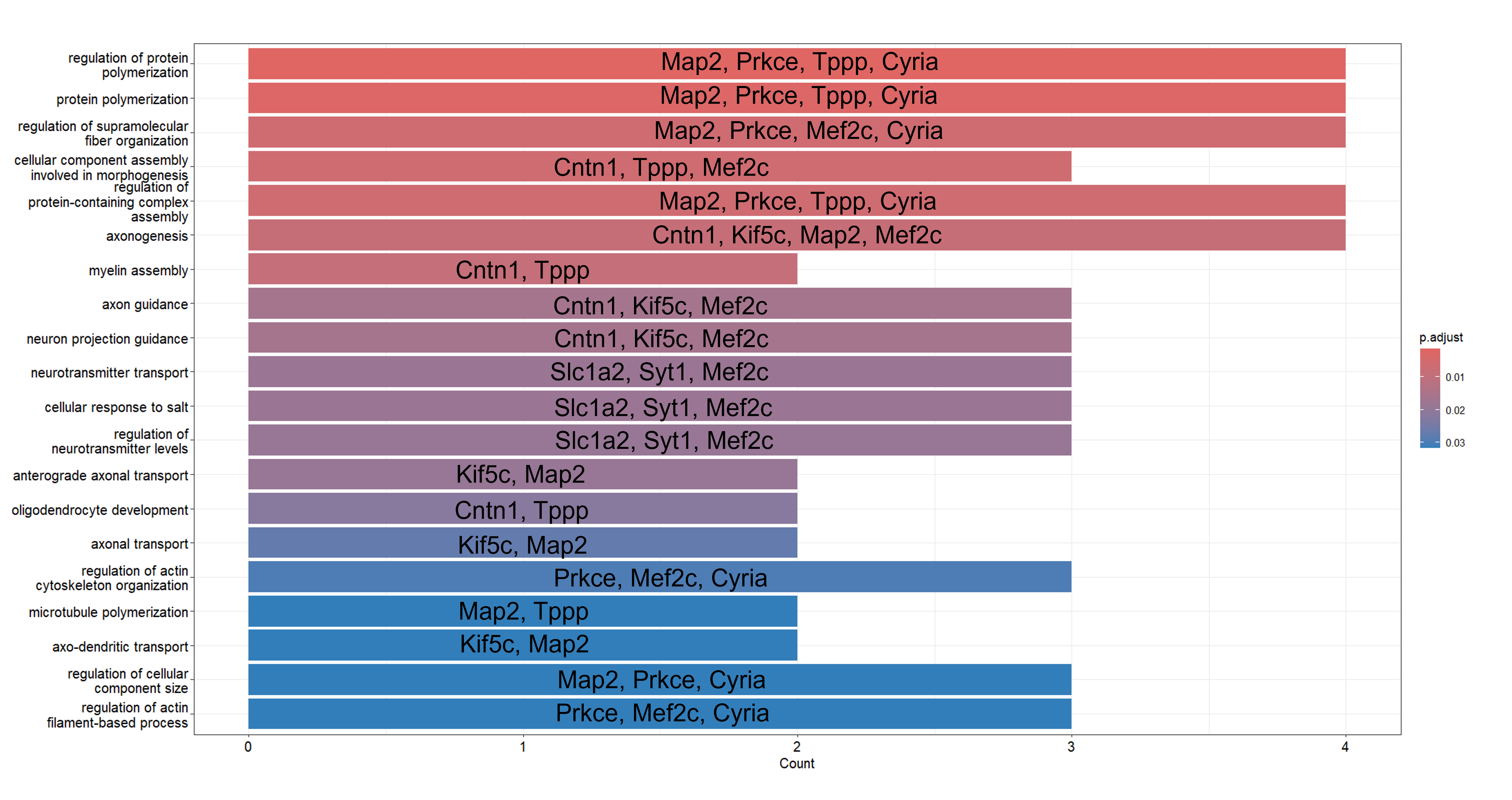

Supplement: Supplementary file 4 — Supplementary file4 (PNG 953 KB) [file 12035_2024_4113_MOESM4_ESM.png]
